# Aptamers: An Emerging Tool for Diagnosis and Therapeutics in Tuberculosis

**DOI:** 10.3389/fcimb.2021.656421

**Published:** 2021-07-01

**Authors:** Shruti Srivastava, Philip Raj Abraham, Sangita Mukhopadhyay

**Affiliations:** ^1^ Laboratory of Molecular Cell Biology, Centre for DNA Fingerprinting and Diagnostics (CDFD), Hyderabad, India; ^2^ Unit of OMICS, ICMR-Vector Control Research Centre (VCRC), Puducherry, India

**Keywords:** aptamer, Systematic Evolution of Ligands by Exponential Enrichment, *Mycobacterium tuberculosis*, tuberculosis, diagnosis, therapeutics

## Abstract

Tuberculosis (TB) has been plaguing human civilization for centuries, and currently around one-third of the global population is affected with TB. Development of novel intervention tools for early diagnosis and therapeutics against *Mycobacterium tuberculosis* (*M.tb*) is the main thrust area in today’s scenario. In this direction global efforts were made to use aptamers, the chemical antibodies as tool for TB diagnostics and therapeutics. This review describes the various aptamers introduced for targeting *M.tb* and highlights the need for development of novel aptamers to selectively target virulent proteins of *M.tb* for vaccine and anti-TB drugs. The objective of this review is to highlight the diagnostic and therapeutic application of aptamers used for tuberculosis. The discovery of aptamers, SELEX technology, different types of SELEX development processes, DNA and RNA aptamers reported for diseases and pathogenic agents as well have also been described in detail. But the emphasis of this review is on the development of aptamers which can block the function of virulent mycobacterial components for developing newer TB vaccine candidates and/or drug targets. Aptamers designed to target *M.tb* cell wall proteins, virulent factors, secretory proteins, or combination could orchestrate advanced diagnosis and therapeutic measures for tuberculosis.

## Introduction

Tuberculosis (TB) caused by *Mycobacterium tuberculosis* (*M.tb*) is a leading cause of death from a single infectious agent worldwide. World health organization (WHO) estimates that 10 million people died of TB in 2018. In this scenario, development of novel approaches for early diagnosis and therapeutics against *M.tb* is main thrust area. Since conventional control measures have been partially successful in keeping TB epidemic under check, identification of biomolecules which can block the function of virulent mycobacterial components is crucial for developing newer TB vaccine candidates and/or drugs. In this context, aptamers, also known as ‘chemical antibodies’, that specifically recognize *M.tb* or inhibit the function of its virulent proteins are being developed (Chen et al., 2007). The aptamers are reported 30 years ago in 1990 (Tuerk and Gold, 1990; Ellington and Szostak, 1992). Aptamers are single-stranded DNA or RNA oligonucleotides that are capable of binding target molecules with high specificity and affinity. Structurally, they are relatively small biomolecules (ranging from 20 to 60 nucleotides) and mimic antibodies as they specifically bind to their targets. In comparison to antibodies, they have shorter generation time, lower manufacturing cost, higher modifiability, better thermal stability, higher target potential, and most importantly, no batch-to-batch variability. Due to multiple advantages over antibodies, they are being used as diagnostics, biosensors, and targeted therapeutics (Zhang et al., 2018) and are touted as a replacement for the use of antibodies in ELISA (Toh et al., 2015). In this article, we review the discovery of aptamers with special emphasis on how they are useful for diagnosis and therapeutic purposes against *M.tb*.

## SELEX: An Art of Aptamer Synthesis

**Figure 1:**
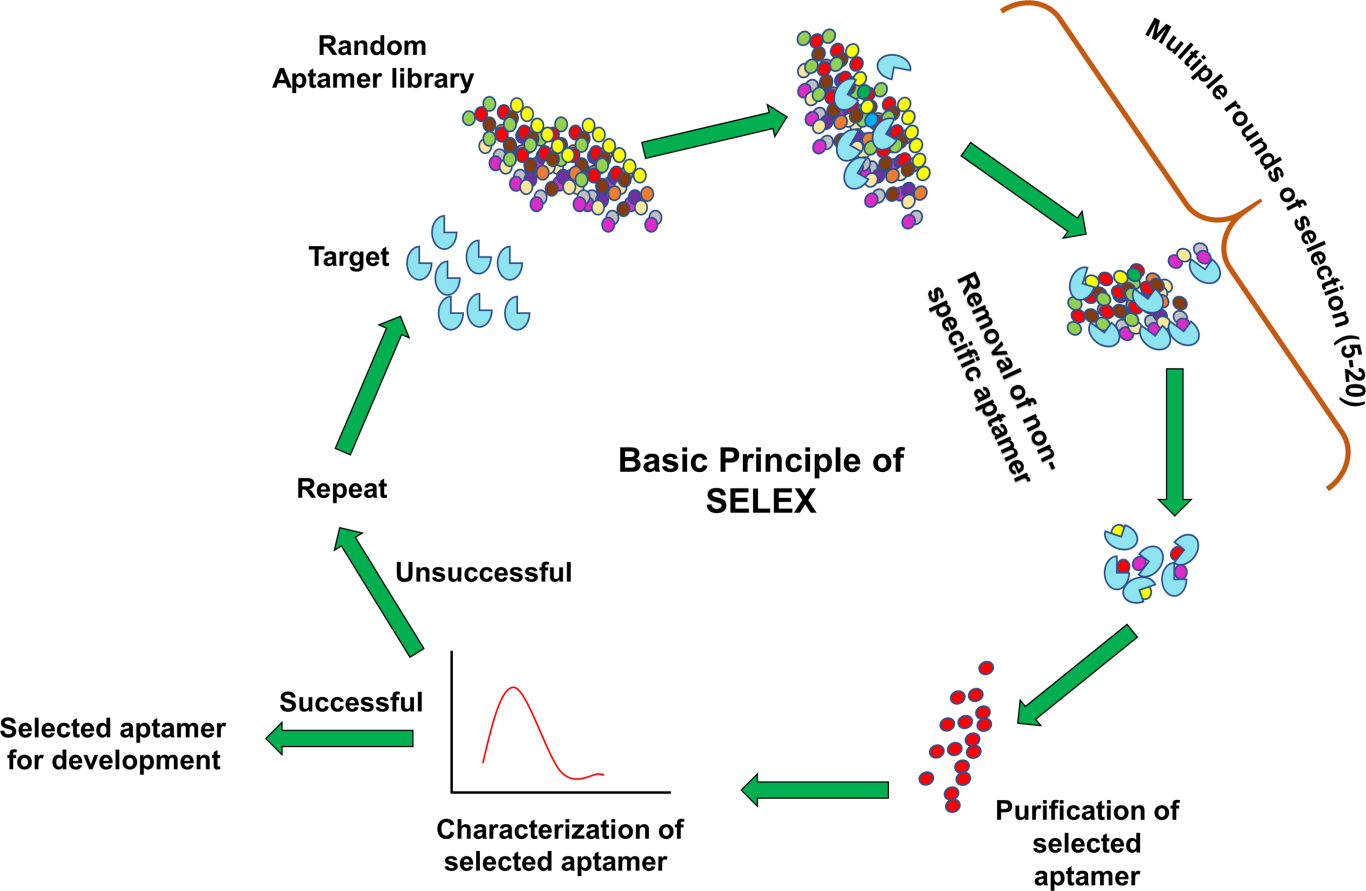
Systematic evolution of ligands by exponential enrichment (SELEX) procedure. A library of aptamers with random oligonucleotides is incubated with target. Unbound aptamers are washed off during multiple round selection. The specific aptamer is enriched from the pool and subjected to various bioanalytical and biological assays. Aptamers are either further developed for several applications like therapeutics and diagnostics (successful) or again feed into the same SELEX cycle (unsuccessful).

**Figure 2:**
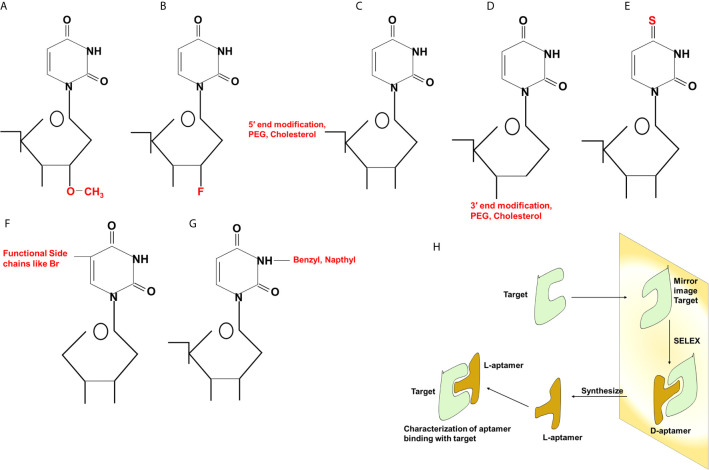
Different types of modifications in aptamers. Chemical modifications at 2′ position **(A, B)**, 5′ and 3′ **(C, D)** ends of the sugar component, replacement of oxygen (O) with sulfur (S) **(E)**, incorporation of functional chains **(F)** or benzyl or naphthyl group **(G)** at the nitrogenous base. Generation of Spiegelmers **(H)**. Aptamers are generated against target having mirror image configuration. Aptamers that bind to mirror image configuration of target are selected, PEG, polyethylene glycol; Br, bromine; F, fluorine.

Aptamers can be synthesized in large amounts as they are structurally stable for longer storage without or with minimal loss in activity. The procedure of *in vitro* synthesis of aptamers is known as “Systematic Evolution of Ligands by Exponential enrichment (SELEX)” (Stoltenburg et al., 2007). Aptamer generation is a long and exhaustive process. Conventionally, an oligonucleotide library contains a pool of 50−90 single-stranded random nucleotide sequences bordered by primer binding sites flanking at both ends. The mechanism of aptamer generation involves the following steps: **(i)** generation of random library of 1014−1016 single stranded oligonucleotides, **(ii)** incubation of oligonucleotides with its target, **(iii)** separation of bound oligonucleotides from unbound ones, **(iv)** selection of specific oligonucleotides, amplification by PCR (DNA aptamers) or RT−PCR (RNA aptamer), and **(v)** finally characterization of aptamer by sequencing (**Figure 1**). For the synthesis of RNA aptamer library, single-stranded DNA library having T7 RNA polymerase promoter sequence at 5′-region is generated. Such single-stranded DNA library is converted to double-stranded DNA, and *in vitro* transcription is performed to generate the desired RNA aptamers. All the steps are repeated till the desired oligonucleotide (or aptamer) with high binding affinity is obtained. Once the desired clones are obtained, they are further optimized to maximize the function. They are truncated or reduced in size to achieve minimal aptamer length with maximum binding affinity for the target. The preferred optimal length for aptamers is 15−45 nucleotides with molecular weight of ∼5−15 kDa. Aptamers bind to their targets with pico to micromolar binding affinity. In recent times, various types of SELEX processes have been developed for specific purposes (**Table 1**). SELEX process-generated aptamers are non-modified, and they are further subjected to various modifications at sugar moiety, phosphate modifications, nucleoside modification, and capping modifications. Some of the examples of modifications are; 2′-fluoro (2′-F) ribose, 2′-amino (2′-NH2) ribose, 2′-O-methyl (2′-OMe) ribose (**Figure 2**) (Maier and Levy, 2016). “Slow off-rate modified aptamers-(SOMAmers)” are new class of aptamers where deoxyribose thymine (dT) bases are replaced by deoxyribose uridine (dU) base at 5′ position in the heterocyclic ring in oligonucleotide pool. Several replacements can be made at 5′ position to generate a vast range of aptamers with different binding affinity and kinetics properties increasing the possibilities of finding suitable aptamer (Maier and Levy, 2016). Naturally occurring nucleotides are D-oligonucleotide, and they form right-handed helix. Mirror image aptamers (spiegelmers) are L-oligonucleotides, and they form left-handed helix. For spiegelmer generation, first conventional D-oligonucleotides are selected against mirror-image target. The selected D-oligonucleotides are chemically synthesized in reverse configuration as L-oligonucleotides (**Figure 2**) (Vater and Klussmann, 2015). Suitable modifications can be incorporated either during selection process (SELEX) or post-selection (post-SELEX) step. Non-modified aptamers are generally less stable and immunogenic than modified aptamers. Modifications prevent aptamers from nuclease-mediated degradation, increase binding affinity, and allow coupling with other molecules, drugs, or nanoparticles (Smith and Zain, 2019). After initial modification, aptamers go through several analytical assays for the assessment of the effect of modification on their binding affinity with target. Final aptamer product is different from the initial one and may possess single or multiple modifications in its structure. Aptamers are studied extensively for their pharmacological kinetics, toxicological feature, metabolism, and physiological clearance (Li et al., 2017; Smith and Zain, 2019).

**Table 1:** Types of SELEX development processes.

S. No.	Type	Description	Reference
**1.**	Negative SELEX	For removal of non-specific aptamers	Ellington and Szostak, 1992
**2.**	Counter SELEX	For removal of cross-reactive aptamers by incubating them targets obtained from related species	Jenison, 1994
**3.**	Genomic SELEX	For the identification of binding motif present in the genome of an organism	Singer et al., 1997
**4.**	*In vivo* SELEX	Aptamers are generated *in vivo*, inside the cell and then characterized	Coulter et al., 1997
**5.**	Chimeric SELEX	In this method, well- characterized aptamers are fused together so that the resulting aptamer can bind to different targets (one aptamer can recognise two or more different targets)	Burke and Willis, 1998
**6.**	Cell-SELEX	For the development of aptamers which recognise markers present in whole cell	Homann and Goringer, 1999
**7.**	Indirect SELEX	In this SELEX method, the binding of aptamers with their targets are metal-ion dependent	Kawakami et al., 2000
**8.**	Photo- SELEX	In this method, nucleotides are light-sensitive and irradiation with UV rays is employed to select the specific aptamer-target from the pool	Golden et al., 2000
**9.**	Toggle SELEX	For identification of cross- reactive aptamers by using toggled targets	Bianchini et al., 2001
**10.**	Tailored SELEX	Aptamers often contain primer- hybridization site. Introduction of cleavable primer hybridization site in aptamers will select for primer-free-aptamers.	Vater et al., 2003
**11.**	CE (Capillary electrophoresis)- SELEX	This process selects high affinity aptamers in a few cycles (2-4) thus shortens the aptamer selection process	Mendonsa and Bowser, 2004; Mosing and Bowser, 2009
**12.**	FluMag SELEX	Targets are tagged with fluorophores	Stoltenburg et al., 2005
**13.**	Target expressed on cell surface- SELEX (TECS- SELEX)	In this method, a cell is engineered to express a recombinant protein which will be used as target protein for aptamer development	Ohuchi et al., 2006
**14.**	Nanoselection based SELEX	One step method to isolate aptamers using fluorescence and atomic force microscopy	Peng et al., 2007
**15.**	MonoLEX	Column chromatography and pyrosequencing is used to select specific aptamer sequences	Nitsche et al., 2007
**16.**	Microfluidic SELEX	Selection of aptamers are performed on a microfluidic chip	Cho et al., 2010.
**17.**	High- throughput SELEX	In this method, high-throughput DNA sequencing and advanced bioinformatic analysis is coupled for aptamers selection	Hoon et al., 2011
**18.**	Particle display SELEX	Flow cytometry based method for aptamer selection	Wang et al., 2014
**19.**	Hi-fidelity SELEX	Digital-PCR is used to intensify the SELEX selection and development	Ouellet et al., 2015
**20.**	Isogenic cell SELEX	Aptamers are first selected against targets overexpressed on isogenic cell line, then counter- selected against microRNA mediated silencing of targets	Takahashi et al., 2016

## Applications of Aptamer

Applications of SELEX and aptamer platform can be used for both diagnosis and drug delivery. In recent times, several research articles have reported the generation of aptamers against microbial pathogens and diseases (**Table 2**) (Cho et al., 2011; Hong and Sooter, 2015; Nimjee et al., 2017). *Trypanosama cruzi* is a blood-borne parasite and known to cause Chagas disease. RNA aptamer generated using whole cell SELEX method was able to detect *T. cruzi* trypomastigotes in the blood (Nagarkatti et al., 2012). Aptamers can easily be coupled with magnetic bead and fluorescent molecule. Exploring this ability, DNA aptamer-based sandwich assay was developed to detect soluble protein of *Leishmania* major (Bruno et al., 2014). Plasmodium lactate dehydrogenase (PLDH) and *Plasmodium falciparum* glutamate dehydrogenase (PfGDH) convert a blue-dye Resazurin to Resorufin (pink). Magnetic bead-coated aptamers were developed to capture and separate PLDH, PfGDH from serum. The conversion from blue to pink color was measured using either spectrophotometer or by qualitative visual interpretation. This is a low cost, portable, user-friendly diagnostic test (Singh et al., 2019). *Salmonella* sp. contamination is responsible for food-borne infections. Therefore, the detection of *Salmonella* sp. in contaminated food is a critical preventive measure in public health. By coupling 6-carboxyfluorescein (FAM), 27-nucleotide aptamer (LA27) was developed to detect LPS from *Salmonella typhimurium* (Ye et al., 2019). Also, aptamer-modified magnetic multifunctional nanoprobe (APT-FMNP) was developed to detect *S. typhimurium* in milk, serum, and urine (Li et al., 2018). DNA-based aptamer to detect tularemia antigen from different subspecies of *Francisella tularensis* was designed at the Air Force research laboratory of USA. It was found that novel anti-tularemia aptamer cocktail can be used as a detection reagent for a potential biological warfare agent like *F. tularensis* (Vivekananda and Kiel, 2006).

**Table 2:** Aptamers reported for microbial pathogens and diseases.

S. No.	Target	Diseases or Pathogenic agent	References
**DNA aptamer**			
**1.**	AS1411	Acute myeloid leukemia	Ireson and Kelland, 2006
**2.**	SARS-CoV N protein	Pathogenic agent	Cho et al., 2011
**3.**	*E. coli* K88	Pathogenic agent	Hong and Sooter, 2015
**4.**	*Salmonella* Paratyphi A	Pathogenic agent	Hong and Sooter, 2015
**5.**	*Listeria monocytogenes*	Pathogenic agent	Hong and Sooter, 2015
**6.**	*Shigella dysenteriae*	Pathogenic agent	Hong and Sooter, 2015
**7.**	*Streptococcus mutans*	Pathogenic agent	Hong and Sooter, 2015
**8.**	*Francisella tularensis* subspecies(subsp.) *japonica* bacterial antigen	Pathogenic agent	Hong and Sooter, 2015
**9.**	HIV reverse transcriptase	Pathogenic agent	Hong and Sooter, 2015
**10.**	Dengue virus type-2 envelope protein domain III	Pathogenic agent	Hong and Sooter, 2015
**11.**	HCV envelope surface glycoprotein E2	Pathogenic agent	Hong and Sooter, 2015
**12.**	Avian influenza H5N1	Pathogenic agent	Hong and Sooter, 2015
**13.**	*Mycobacterium* *tuberculosis H37Rv*	Pathogenic agent	Hong and Sooter, 2015
**14.**	ARC1772	Thrombosis	Nimjee et al., 2017
**15.**	PTK7 aptamer	Leukemia	Nimjee et al., 2017
**16.**	Nu172	Anticoagulation	Maimaitiyiming et al., 2019
**17.**	E10030	Age-related macular degeneration (AMD)	Maimaitiyiming et al., 2019
**18.**	N55	Atherosclerosis	Sola et al., 2020
**19.**	GBI-10	In several types of tumors	Sola et al., 2020
**20.**	SQ-2	Pancreatic ductal adenocarcinoma (PDAC)	Sola et al., 2020
**RNA aptamer**			
**21.**	A9 and A10	Prostate cancer	Nimjee et al., 2017
**22.**	RNA 14-16 against p68, helicase	Liver metastasis	Nimjee et al., 2017
**23.**	Pegaptanib	AMD	Nimjee et al., 2017
**24.**	ARC1905	AMD	Maimaitiyiming et al., 2019
**25.**	ARC19499	Hemophilia	Maimaitiyiming et al., 2019
**26.**	NOX-H94	Anemia	Maimaitiyiming et al., 2019
**27.**	EYE001	AMD	Maimaitiyiming et al., 2019
**28.**	MRP1Apt	Melanoma cancer stem cells	Sola et al., 2020
**29.**	A1	Breast cancer cells	Sola et al., 2020
**30.**	G-3	HIV infection blockade	Sola et al., 2020
**31.**	P30-10-16	Influenza B virus infection blockade	Sola et al., 2020
**32.**	PB	Prostate cancer	Sola et al., 2020
**33.**	GL21.T	Axl-dependent cancers	Sola et al., 2020
**SOMAmer: Slow-Off-rate-Modified-Aptamer**			
**34.**	*Clostridium* *difficile* binary toxin (CdtA)	Pathogenic agent	Ochsner et al., 2014
**35.**	Proprotein convertase subtilisin/kexin type 9	Disease	Gawande et al., 2017
**36.**	Vaccine antigen in the human papillomavirus (HPV) vaccine Gardasil	Pathogenic agent	Trausch JJ et al., 2017
**37.**	Glypican-3 SOMAmer	Hepatocellular carcinoma (HCC)	Duo et al., 2018
**38.**	*Mycobacterium* *tuberculosis*	Pathogenic agent	Golichenari et al., 2018

The first aptamer approved for use as therapy in humans was a RNA-based molecule (macugen, pegaptanib) which is administered locally to treat age-related macular degeneration (AMD) by targeting vascular endothelial growth factor. Macugen received FDA approval in December 2004 for the treatment of AMD (Ng et al., 2006). RB006 aptamer binds to factor IXa and specifically blocks the conversion of factor X to factor Xa (Nimjee et al., 2005). Conversion of factor X to factor Xa is an important step in prothrombin assembly and thrombin generation. Hemophilia is a genetic disorder which causes defective intrinsic blood coagulation pathway. In hemophilia, coagulation factor VII and factor IX are deficient. Aptamer ARC19499 binds to tissue factor pathway inhibitor (TFPI), a negative regulator of extrinsic pathway, allowing the initiation of extrinsic coagulation pathway (Waters et al., 2011). AXL is a tyrosine kinase receptor overexpressed in solid tumor types. Aptamer GL21.T conjugated with an anti-tumor microRNA, let-7g, was injected *via* intravenous route in mice. A decrease in tumor growth was noticed in mice with A549 (Axl^+^)-Luc tumor xenograft (Esposito et al., 2014). Cytotoxic T cell antigen-4 (CTLA-4) is a cell surface receptor that decreases the immune reaction against tumors. Tetramer-RNA aptamer could inhibit CTLA-4 function in mice B16/F10.9 melanoma allograft model when injected intraperitoneally (Santulli-Marotto et al., 2003).

It is known that human immunodeficiency virus (HIV) attacks CD4^+^ T-cells, macrophages, and dendritic cells. An incapacitated immune system fails to protect humans and leads to development of acquired immunodeficiency syndrome (AIDS). Gp120 is a glycoprotein of HIV virus that binds to CD4^+^ T-cell receptor and invades the host. A novel dual inhibitory function-based anti-gp120 aptamer-siRNA chimera (A-1 and B-68) could bind to gp120, thus preventing the viral entry in CHO cell line (Zhou et al., 2011). A modified thioaptamer, R12-2, could specifically bind to HIV-1 RNaseH and inhibit viral replication in cell culture (Somasunderam et al., 2005). Ebola virus causes life-threatening hemorrhagic fever. Interferons mediate anti-viral function in humans, and VP35 protein of EBOLA inhibits the production of interferon regulatory factor 3 (IRF-3). Aptamers (1G8-14 and 2F11-14) could bind to interferon inhibitory domain (VP35IID) of VP35 protein, preventing the inhibition of IRF-3 *in vitro* (Basler et al., 2003; Cardenas et al., 2006). *Staphylococcus aureus* causes staphylococcal toxic shock syndrome (TSS), food-borne infection, and hospital-acquired infection. Enterotoxin B (SEB) from *S. aureus* activates T cells to generate cytokine storm. Aptamer A11 was developed to inhibit SEB activity, preventing cytokine storm (Wang et al., 2015). Anthrax disease is caused by a toxin secreted from *Bacillus anthracis*. An important component of anthrax toxin is a lethal factor (LF) that has protease activity. LF cleaves and activates mitogen-activated protein kinase kinase (MAPKK). ssDNA aptamer (ML12) prevents the protease activity of LF reducing its toxicity. It is being considered as a potential drug target against LF (Lahousse et al., 2018).

In addition, aptamers are also discovered as anti-cancer agents and used in the treatment of neurological disorders. One of the most promising aptamer, AS1411, induces growth inhibition *in vitro* and has shown activity against human tumor xenografts *in vivo* (Ireson and Kelland, 2006). Nucleolin is an external membrane protein over-expressed in cancerous cells. Aptamer AS1411 could bind to nucleolin, inhibiting its activity (Girvan et al., 2006; Soundararajan et al., 2008). AS1411 was the first drug identified to target nucleolin and used in the clinical trial of cancer treatment (Mongelard and Bouvet, 2010). Target specificity is an important trait of anti-cancer therapeutic. Prostate-specific membrane antigen (PSMA) is a biomarker for solid tumors. RNA aptamers (A9 and A10) against PSMA were identified. A polymeric nanoparticle with docetaxel drug–aptamer complex was able to reduce tumor growth in xenograft mice when injected intraperitoneally (Dassie et al., 2009). CT26 is a colon cancer cell line. In an effort to identify, specifically located aptamer, mice bearing intrahepatic tumor were injected Cy3-labeled RNA aptamers. Aptamers 14–16 were found to be localized in intrahepatic CT26 tumor tissues (Mi et al., 2010). Blood–brain barrier is major obstacle in the treatment of neurological diseases. Bioavailability of drugs is restricted in the brain. Aptamer pool of random RNA sequences was injected into tail vein of mice, and the localization of aptamers was checked in the brain. Aptamer A15 showed specific localization in the brain (Cheng et al., 2013). Aptamers are used for inhibition of biofilm formation, microbial toxins, and also as anti-bacterial agents. The use of aptamers for therapeutic purpose often encounters several roadblocks like stability, renal clearance, or filtration, elimination of excess un-bound aptamers, biodistribution, or bioavailability at the desired tissue locations (Kovacevic et al., 2018; He et al., 2020).

**Figure 3:**
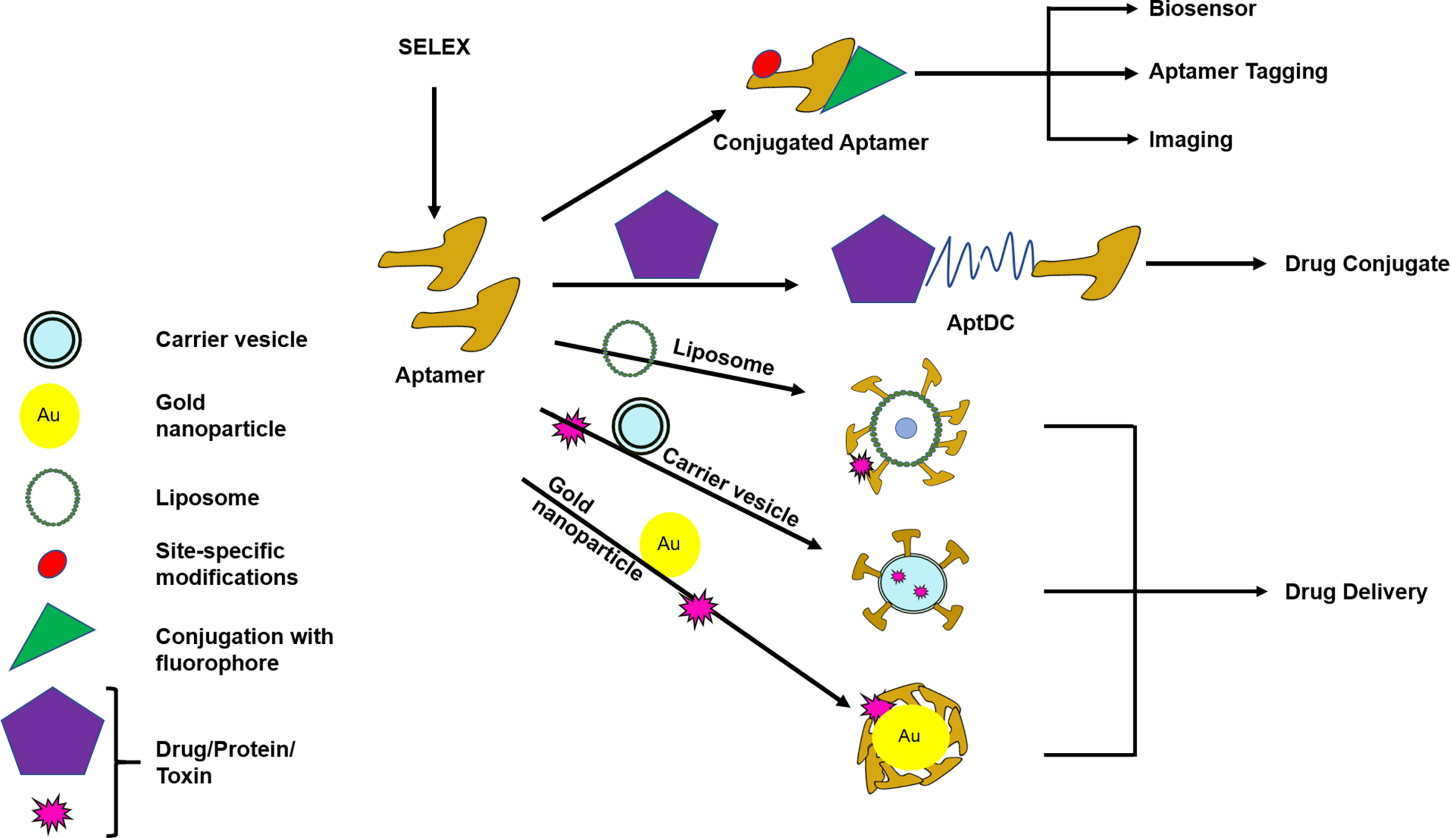
Modification and conjugation of aptamers for the use of diagnostic and/or therapeutic purpose. Aptamers can be modified or conjugated with different substances to carry out various functions like drug delivery, drug conjugate, imaging, biosensor, or aptamer tagging. AptDC–Aptamer–Drug-conjugate. Routes of drug delivery are oral, intramuscular, parenteral, intravenous, rectal, topical, otherwise specified. Specialized drug delivery modes are liposomal, nanoparticle, carrier vesicle. Drug delivery optimization is needed to obtain the desired effect.

Modifications in aptamers enable them to overcome the above mentioned hurdles and impart flexibility to execute various functions like: drug delivery, drug conjugation, imaging, biosensor, or aptamer tagging (especially useful for RNA aptamer) (**Figure 3**). In the current COVID-19 scenario, aptamers are being explored for the diagnosis as well as treatment options world-wide. In a recent article, aptamers have been reported against receptor-binding-domain (RBD) which could prevent binding of RBD with ACE2 receptor reflecting the potential of aptamers in SARS-CoV-2 (severe acute respiratory syndrome coronavirus 2) virus diagnosis and treatment (Song et al., 2020). Further, an aptamer that could bind to viral nucleoprotein, nucleolin, has been developed as a proof-of-concept for SARS-CoV-2 detection (Thomas, 2020). N protein of SARS-CoV-2 is essential for viral genome assembly; thus aptamer against N protein of SARS-CoV-2 might extend its use in diagnosis and treatment simultaneously (Song et al., 2020). This is a standout feature where an aptamer serves the purpose of both diagnosis and treatment.

## Challenges in Tuberculosis Diagnosis and Treatment

Despite extensive research activities, *M.tb* continued to kill millions of humans every year. Limitations of rapid and accurate diagnosis of tuberculosis, emergence of drug-resistance and HIV-TB co-infections are complicating TB treatment. For TB diagnosis, smear microscopy is a less sensitive test. Culturing *M.tb* is the most accurate method to identify the pathogenic species, but it is time consuming and not suitable for point-of-care (POC) diagnosis. In PCR-based diagnosis, infection is confirmed by the presence of mycobacterial DNA or RNA sequence; however, the thermal cyclers are not readily available in resource-limited settings. On the other hand, rapid and less expensive POC, such as serological assays, is banned for testing the TB. WHO endorsed Gene-Xpert MTB/RIF, a specific, fast technique and revolutionary TB detection method based on PCR-DNA amplification, but it has certain limitations (WHO, 2019). Gene-Xpert fails to detect TB in children, smear-negative-TB infection. Also, for accuracy and fast performance, Gene-Xpert *M.tb* requires skilled manpower and sophisticated instrument setup. Tuberculin skin test (TST) and Interferon-*γ*-Release-Assay (IGRA) are immunological tests based on the principle of antigen–antibody reaction. Interferon-*γ* is a pro-inflammatory cytokine, released during tuberculosis infection. IFN-*γ* is measured by ELISA from TB patients’ blood. These tests fail to differentiate between active TB, latent TB, or relapsed TB. Despite these advancements, the diagnosis of *M.tb* infection still requires a rapid, accurate, and efficient diagnostic platform. It has been estimated that 0.5 million people fell ill with drug resistant TB, and only one in three TB patients had access to treatment (WHO, 2019). There is a requirement of USD 10.1 billion for TB diagnosis, treatment, and care. Successful treatment of tuberculosis (TB) infection is one of the fierce challenges faced by physicians, which is mainly because of the unavailability of accurate information about *M.tb* species and its drug-resistant status leading to treatment failure. As a result, multi-drug-resistant (MDR) and extensively drug resistant (XDR) *M.tb* arise due to over and improper use of antibiotics during the treatment of TB (WHO, 2019). In the current COVID-19 crisis, it has been estimated that the death toll due to TB may rise up to 20% over the next five years (Hogan et al., 2020) and TB treatment may be side-lined and adversely affected.

## Aptamers in the Diagnostic and Therapeutic Applications of *M.tb*

**Figure 4:**
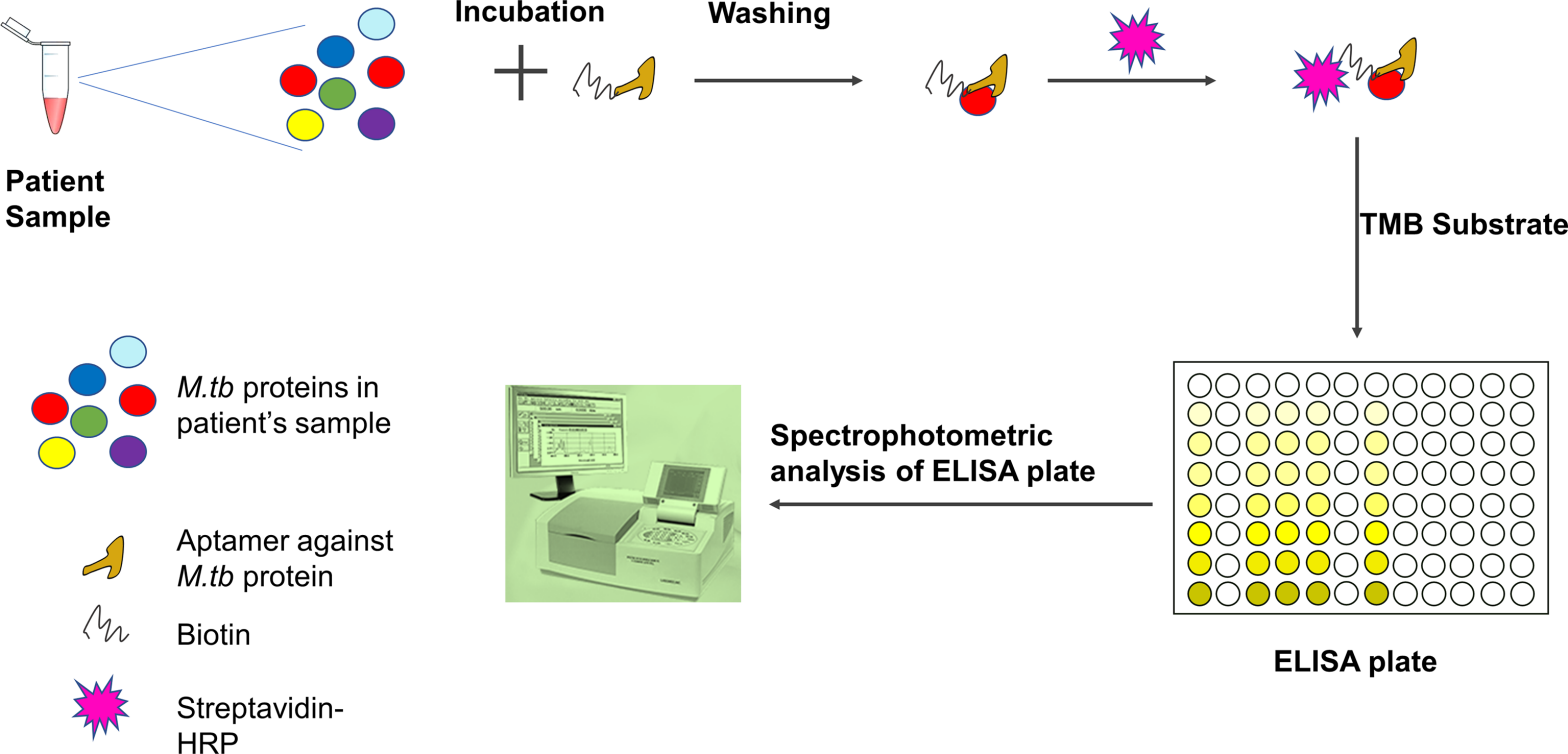
Detection of *M.tb* proteins using aptamer-based ELISA or ELONA (ELONA-enzyme-linked oligonucleotide assay). In an ELISA plate, the patient sample is incubated with biotinylated aptamer specific for *M.tb* protein. After washing, streptavidin-HRP is added and ELISA is developed using TMB substrate. The optical density is measured using a spectrophotometer. Patient sample could be blood, serum, sputum, or body fluid. Viscous fluid like sputum can be diluted and vortexed before incubation with aptamer.

Global efforts were taken to introduce aptamers for rapid diagnosis and therapeutic applications for tuberculosis (Zhu et al., 2012; Golichenari et al., 2018; Li et al., 2018). A gold nano-particle conjugated-CFP10-ESAT6 DNA aptamer was developed to detect *M.tb*, and it could differentiate between pathogenic and non-pathogenic *M.tb* bacteria (He et al., 2016). ESAT-6-based aptasensor ESAT6-P-MOF-rGO (metal organic framework graphene oxide-MOFGO) could detect ESAT-6 secretion in human serum samples (Li et al., 2018). Similarly, a sandwich ELISA can detect MPT64 aptamer (**Figure 4**) (Zhu et al., 2012). A pro-inflammatory cytokine, IFN-*γ* is secreted in response to mycobacterial antigens. Recently, for detection of IFN-*γ* release, a SPR based-dual-aptamer sensor has been designed. In this sensor, aptamer against IFN-*γ* is coupled with streptavidin specific aptamer. Streptavidin aptamer performs dual function of “a reporter” and “an amplifier”. Such dual DNA probe was able to detect low level of IFN-*γ* in plasma isolated from healthy individuals because they have less IFN-*γ* concentration (Min et al., 2008; Chang et al., 2012). Another aptamer, MTB36 also shows preferential binding with *M.tb* over *M*. *bovis* BCG suggesting its ability to discriminate between close species (Mozioglu et al., 2016). PPK (polyphosphate kinase gene) regulates inorganic polyphosphate (polyP) function and aids in virulence. An aptamer G9 has been shown to inhibit PPK activity rendering inhibition of polyp-mediated metabolic process in *M.tb* (Shum et al., 2011). Culture filtrate protein-10 (CFP-10) and early secreted antigenic target of 6 kDa (ESAT-6) proteins are secreted during TB infection. Aptamer CSIR2.11 could detect CFP-10-ESAT-6 complex as well as CFP-10 alone from sputum sample of TB patients. The sensitivity and specificity of CSIR 2.11 were found to be 100 and 68.75% in sputum sample of TB patients (Rotherham et al., 2012). A DNA aptamer, H63SL2-M6 against HspX, a mycobacterial antigen highly expressed in TB patients, had been shown to detect HspX in sputum of TB infected individuals using aptamer-linked immobilized sorbent assay (Aptamer ALISA). The performance of ALISA was superior to conventional ELISA (Lavania et al., 2018). Further, an electrochemical sensor (ECS) device based on H63SL2-M6 aptamer was developed as POC diagnosis (Lavania et al., 2018). HupB (Rv2986c) is a promising drug target against TB. It is an essential histone-like protein of *M.tb* which protects *M.tb* DNA from damage and regulates iron homeostasis (Pandey et al., 2014). G-quadraplex forming DNA aptamers, HupB-4T and HupB-13T, were developed to block DNA-binding activity of HupB. HupB proteins are required for macrophage entry of *M.tb*. Inhibition of HupB function by HupB-4T and HupB-13T has been shown to arrest the bacterial entry into macrophages. Thus HupB-4T and HupB-13T could serve as inhibitors of HupB functions (Kalra et al., 2018). A proteomic-based SOMAscan was developed for *M.tb* diagnosis, but it failed to identify noticeable difference between TB and non-TB patients’ serum (Russell et al., 2017). Malate synthase (MS) enzyme of the glyoxylate pathway converts malate by using glyoxylate and acetyl-CoA. MS expresses in cell wall and promotes adhesion of bacteria. Another g-quadruplex DNA aptamer, MS10, binds to MS and inhibits its enzymatic activity (Dhiman et al., 2019). ESX3 secretion system of *M.tb* secretes EsxG protein. RNA aptamers against G43 and G78 bind to EsxG but not to EsxA, suggesting that aptamers can differentiate between closely related proteins and bind to their specific partners only (Ngubane et al., 2014). Whole-cell-SELEX identifies the receptors present on the target cell in their native confirmation. Hence, aptamers developed in this way perform better than aptamers developed using recombinantly purified target (Dwivedi et al., 2013; Aimaiti et al., 2015). Aptamers generated through such method could differentiate *M.tb* H37Rv from non-tuberculous mycobacteria. An aptamer MA1 or combination of MA1/MA2 aptamers recognizes *M.tb* H37Rv preferentially as compared to *M. marianum* (Aimaiti et al., 2015)*. M.tb* Ag85 complex is secreted as protein complex of three proteins, A, B, and C and is important for cell wall biosynthesis. Ag85 complex is considered to be a potent biomarker for TB diagnosis. Cerebrospinal fluid of patients suffering from tuberculous meningitis also showed the presence of *Mycobacterium* antigen 85 complex (Ag85 complex) (Kashyap et al., 2005). A fluorescent aptasensor, ATTO647N-Apt22, is developed to detect Ag85 complex (Ansari et al., 2017). Entry of aptamers are relatively new in the field of TB drug discovery/or development. Hence, there is only a handful of examples where aptamers have been shown or developed for anti-mycobacterial function (**Table 3**). The *M.tb* virulent proteins infect macrophages and weaken the immune system. Since macrophages are preferred niche for *M.tb*, aptamers preventing macrophage invasion can help in controlling *M.tb* infection (Chen et al., 2012; Kalra et al., 2018). NK2 aptamers inhibit invasion of *M.tb* in macrophages, enhances IFN-*γ* production, and increases survival of mice infected with *M.tb* (Chen et al., 2012). The 6-kDa early secreted antigenic target (ESAT-6) and 10-kDa culture filtrate protein (CFP-10) are secretory proteins of virulent *M.tb.* ssDNA aptamers (CE24 and CE15) can detect CFP-10 and ESAT-6 proteins respectively in serum samples from active pulmonary tuberculosis patients, extrapulmonary TB patients, and healthy individuals (Tang et al., 2014). Mannose-capped lipoarabinomannan (ManLam), a cell wall component of *M.tb*, is important for *M.tb* pathogenesis (Tang et al., 2016). ManLam inhibits antigen presentation function of dendritic cells. Aptamer T9 can detect ManLAM antigen in serum and sputum samples from active pulmonary tuberculosis (aPTB) patients, extrapulmonary TB (EPTB) patients, and healthy donors with >85% specificity and sensitivity. The performance of T9 based enzyme-linked oligonucleotide assay (ELONA) was comparable with the standard T-SPOT.TB test. T9-based ELONA (**Figure 4**) has been touted as improved diagnostic test with potential to detect inactive TB, smear-negative TB, EPTB, and TB with HIV but its inefficiency to differentiate between LTBI and active TB limits its application. Nonetheless, T9-based ELONA is fast and less expensive than T-SPOT.TB test (Tang et al., 2016). An aptamer ZXL1 recognizes acyl and phosphate structures of ManLAM and binds to *M.tb*-ManLAM, not to BCG-ManLAM. ZXL1 suppresses CD11+ expression in dendritic cells and inhibits *M.tb* H37Rv infection in mice and rhesus monkeys. The ability to differentiate between *M.tb*-ManLam *versus* BCG-ManLam acknowledges the potential of ZXL1 as a future candidate for developing tuberculosis vaccine (Pan et al., 2014). Acetoacetate is an important intermediate molecule in essential amino acid synthesis. Acetohydroxy acid synthase (ASHS) has no known functional analogs in humans, which makes it a good drug candidate. ASHS converts pyruvate molecule to acetoacetate. ssDNA aptamers named as *M.tb*-Apt1 and *M.tb*-Apt6 inhibit ASHS activity and are able to kill MDR strain of *M.tb* (Baig et al., 2015). Aptamer BM2 increases the immunogenicity of BCG against virulent *M.tb*, H37Rv infection in monkeys as well as in mice model (Sun et al., 2016). BM2 aptamer targets BCG–ManLAM–CD44 interaction and initiates M1 macrophage-mediated Th1 (T helper 1) T cell immune response (Sun et al., 2016). Thioaptamers (TA) are modified aptamer having thiophosphate ester bond (Somasunderam et al., 2010). CD44 is a conserved receptor present on macrophages. *M.tb* binds to CD44 and enters inside the macrophages (Leemans et al., 2003). Conjugation of CD44-thioaptamers with SMP (discoidal silicon mesoporous microparticles) enhanced the internalization of aptamers and killed *M.tb*-infected macrophages in mice lung (Leonard et al., 2017).

**Table 3:** Aptamers developed for diagnosis and therapeutic applications in tuberculosis.

S. No.	Aptamer	Type	Organism	Target	Reference
** For Diagnosis**					
**1.**	CE24 CE15	DNA	*M.tb* H_37_Rv	CE protein	Tang et al., 2014
**2.**	CSIR 2.11	DNA	*M.tb*	CE protein	Rotherham et al., 2012
**3.**	MPT64-A1	DNA	*M.tb*	MPT64	Zhu et al., 2012
**4.**	G43 G78	RNA	*M.tb*	EsxG protein	Ngubane et al., 2014
**5.**	MA1	DNA	*M.tb*	Whole-bacterium	Aimaiti et al., 2015
**6.**	Aptamer 1	DNA	*M.tb*	Whole-bacterium	Chen et al., 2007
**7.**	Au-IDE/CFP10- ESAT6	DNA	*M.tb*	Whole-bacterium	He et al., 2016
** For therapeutic**					
**8.**	NK2	DNA	*M.tb* H37Rv	Whole-bacterium	Chen et al., 2007
**9.**	ZXL1	DNA	*M.tb* H37Rv	ManLAM	Pan et al., 2014
**10.**	M. tb -Apt1 M. tb -Apt6	DNA	*M.tb*	Acetohydroxyacid synthase	Baig et al., 2015
**11.**	BM2	DNA	BCG	ManLAM	Sun et al., 2016
**12.**	T9	DNA	*M.tb* Beijing strains	ManLAM	Tang et al., 2016
**13.**	CD44-TA-SMP	--	*M.tb*	CD44 receptor	Leonard et al., 2017

## Future Direction for Development of Aptamers for TB Research

As shown by Chen et al., 2020, an aptamer which was generated for detecting N protein of SARS-CoV2, can also be used for the treatment as well. Such rationale can also be adopted while developing aptamers targeting *M.tb* cell wall proteins, virulent factors, secretory proteins, or combination of above factors that could be a substitute of conventional diagnosis approaches. Aptamers generated against whole bacterium are regarded suitable for diagnostic purposes as they can recognize different epitopes present on *M.tb* and detect them in human fluid *i.e.* blood, serum, bronchoalveolar lavage (Chen et al., 2007; Zhu et al., 2012; Rotherham et al., 2012). Aptamers can also be designed against PE/PPE family proteins as they have shown to induce strong antibody responses in smear negative and extrapulmonary TB and also in individuals with latent tuberculosis (Mukherjee et al., 2007; Khan et al., 2008; Tundup et al., 2008; Abraham et al., 2014; Khan et al., 2016; Abraham et al., 2018). Since approximately 10% of *M.tb* genome codes for PE/PPE proteins, it is of interest to explore the possibility of using the PE/PPE based aptamers for TB and latent TB diagnosis. Various studies have shown that PE/PPE proteins can be used as potential markers for serodiagnosis of active TB as well as latent TB infection. Aptamers for PPE proteins such PPE17 (Abraham et al., 2016), PPE2 (Abraham et al., 2014), PPE68 (Xu et al., 2012), PPE42 (Chakhaiyar et al., 2004; Ireton et al., 2010), PPE57 (Zhang et al., 2007), and PPE41 (Choudhary et al., 2003) may be tested for diagnosis of TB. In addition, aptamers for PE proteins such as PE25 **(** Tundup et al., 2008), PE11 (Narayana et al., 2007) and PE35 (Mukherjee et al., 2007) could be explored for diagnostic assessment. Anti-*M.tb* aptamers may also be required to differentiate different strains of *M.tb* that may help in understanding the transmission dynamics of TB in different geographical locations/endemic areas of TB. It will be worth inventing aptamers that can identify drug resistant *M.tb* isolates for prescribing appropriate anti-TB regimen. *M.tb* resides inside macrophages and incapacitates the human immune system. Thus, aptamers can be designed to block, inhibit, or prevent the functions of virulent factors of mycobacteria and may be exploited as therapeutics. For example, our recent studies (Nair et al., 2009; Nair et al., 2011), indicated an important role of the LRR (leucine rich repeat) 11–15 domains of toll like receptor (TLR) 2 in the induction of non-protective IL-10/Th2 response by PPE18 protein of *M.tb* (Nair et al., 2009; Nair et al., 2011); thus aptamers can be designed to block this interaction to specifically increase the protective Th1-type immune response against *M.tb*. Also our recent study indicates that the PPE2 protein of *M.tb* interacts with p67phox in macrophages and inhibits reactive oxygen species (Srivastava et al., 2019). Aptamers to block this interaction may be useful to increase the innate host defense during *M.tb* infection. Similarly, another protein, PE11, is found to be responsible for cell wall architecture of *M.tb*, contributing to *M.tb* virulence (Singh et al., 2016; Rastogi et al., 2017) and can be the target of aptamer research. Also we report that ESAT-6 protein interacts with *β*_2_-microglobulin of host inhibiting class I-mediated antigen presentation and CD8+ T cell function (Sreejit et al., 2014). It is probably important to design the aptamer to specifically block this interaction to improve CD8+ T cell function which is shown to be poorer and/or delayed during *M.tb* infection (Koul et al., 2004).

## Concluding Remarks

Our current knowledge of cellular and molecular interactions between mycobacteria and host immune responses is poor, and the complex pathobiology of tuberculosis presents a significant challenge in vaccine development. Recent advancement in aptamer technology has come up with novel and imaginative ways to develop therapeutic and diagnostic aptamers against important molecular targets of *M.tb*. In the management of tuberculosis, early and accurate diagnosis is the key. Delayed diagnosis and poor treatment lead to the development of drug resistance. Hence, aptamers which can detect early infection stages or detect drug-resistant *M.tb* strain would be effective in the management of TB. In DOTS (directly observed treatment, short-course) therapy for tuberculosis management, a combination of antibiotics is given for a long period of time. *M.tb* develops drug resistance, and thus the currently available drugs generally fail to clear the infection. Most antibiotics are generated against enzymes important for cell wall formation, biofilm synthesis, or DNA replication, and protein synthesis pathway, or they target they metabolic pathway of *M.tb* (Davydova et al., 2016). Aptamers do not target enzymatic pathway directly. They are designed to recognize their target, bind to and inhibit the functions. In this way, aptamers are believed to overcome the problem of generation of resistant mycobacteria. Aptamers are sensitive to nuclease-mediated degradation. Being smaller in size, localization of aptamers to its site-of-action is challenging. Additionally, aptamers are rapidly cleared from the body due to high renal filtration. There is a new class of aptamers known as SOMAmer (Slow-Off rate-Modified-Aptamer) that are modified nucleotide aptamers having side-chains to facilitate strong hydrophobic interaction with their target. SOMAmer stays in circulation for longer duration. Though aptamers offer a bundle of advantages, they have some shortcomings which need to be overcome before aptamers can be fully exploited true to their potential. In TB diagnosis platform, even highly advanced technique suffers due to extremely low concentration of *M.tb* antigens in serum, blood, urine, *etc*. Though there have been significant improvement in the diagnosis and treatment of tuberculosis, still we are unable to address the basic question of preventing the emergence of drug-resistant *M.tb* or minimizing the side effects of antibiotics or development of an effective anti-TB vaccine. Aptamers are unique, less expensive, and simple oligonucleotide molecules. They are flexible and they can be conjugated with a variety of agents like siRNA, drugs, and nanoparticle molecules (Jiang et al., 2015; Davydova et al., 2016). Advancement in aptamer technology which will certainly lead to selectively target virulent proteins of *M.tb* would pave the way for new and improved vaccine and drug development. Aptamer technology is in its nascent phase; however measured interventions in aptamer have the potential to replace conventional way of TB management in future.

## Author Contributors

SS—Preparation of first draft and revision. PRA—Conceptualization and review. SM—Conceptualization, review and finalization of the manuscript. All authors contributed to the article and approved the submitted version.

## Funding

This work was supported by grants from the Department of Biotechnology (DBT), Government of India (BT/PR20669/MED/29/1072/2016), Science and Engineering Research Board (SERB), Department of Science and Technology (DST), Government of India (DST/SERB/CRG/2019/000239), TATA Innovation Fellowship, DBT (BT/HRD/35/01/03/2018), Council of Scientific and Industrial Research (CSIR), Govt. of India (27(0364)/20/EMR-II), and a core grant from the Centre for DNA Fingerprinting and Diagnostics by the Department of Biotechnology (DBT) to SM.

## Conflict of Interest

The authors declare that the research was conducted in the absence of any commercial or financial relationships that could be construed as a potential conflict of interest.
